# *In Silico* Classification of Proteins from Acidic and Neutral Cytoplasms

**DOI:** 10.1371/journal.pone.0045585

**Published:** 2012-09-26

**Authors:** Yaping Fang, C. Russell Middaugh, Jianwen Fang

**Affiliations:** 1 Applied Bioinformatics Laboratory, The University of Kansas, Lawrence, Kansas, United States of America; 2 Department of Pharmaceutical Chemistry, The University of Kansas, Lawrence, Kansas, United States of America; UMR-S665, INSERM, Université Paris Diderot, INTS, France

## Abstract

Protein acidostability is a common problem in biopharmaceutical and other industries. However, it remains a great challenge to engineer proteins for enhanced acidostability because our knowledge of protein acidostabilization is still very limited. In this paper, we present a comparative study of proteins from bacteria with acidic (AP) and neutral cytoplasms (NP) using an integrated statistical and machine learning approach. We construct a set of 393 non-redundant AP-NP ortholog pairs and calculate a total of 889 sequence based features for these proteins. The pairwise alignments of these ortholog pairs are used to build a residue substitution propensity matrix between APs and NPs. We use Gini importance provided by the Random Forest algorithm to rank the relative importance of these features. A scoring function using the 10 most significant features is developed and optimized using a hill climbing algorithm. The accuracy of the score function is 86.01% in predicting AP-NP ortholog pairs and is 76.65% in predicting non-ortholog AP-NP pairs, suggesting that there are significant differences between APs and NPs which can be used to predict relative acidostability of proteins. The overall trends uncovered in the study can be used as general guidelines for designing acidostable proteins. To best of our knowledge, this work represents the first systematic comparative study of the acidostable proteins and their non-acidostable orthologs.

## Introduction

Protein acidostability is a common problem in biopharmaceutical and other industries because the vast majority of native proteins are only stable in near-neutral pH conditions [1], [2] and exposure of a protein to an acidic environment can cause a loss of activity and denaturalization quickly [3]. In fact, one major reason that most peptide and protein drugs cannot be delivered via the oral route is the strong acidic environment in the gastrointestinal (GI) tract [4], even though the oral route is preferred to the current parenteral administration because it is non-invasive and convenient for self-administration. Therefore it is highly desirable to develop acidostable peptide and protein drugs. In addition, acidostable proteins are also very useful in many other industries such as paper and pulp, oil, food, and environment cleanup, etc.

Some research has been conducted into investigating the factors important to acidostability of proteins in order to be able to design acidostable proteins. For example, Kelch et al studied the folding rates of two proteins in different pH conditions and then compared the sequences of the two proteins [1]. Another study was to analyze the structural properties of proteins with different acidostability [5], [6]. Several studies investigated the pH-dependent property of protein by calculating the pka values based on the three-dimensional structure of protein [7], [8], [9], [10]. Despite these efforts, the mechanisms of protein acidostabilization are far from being fully understood because of a lack of systematic studies. One main reason was that the number of known acidostable proteins was quite limited, which in turn hinders comprehensive studies. A vast majority of natural proteins are unstable in acidic conditions because most organisms live in neutral or near-neutral conditions. Despite some bacteria and archaea that can thrive in acidic environments (i.e. acidophiles), the cytoplasm of most of these organisms is at or close to neutrality [2], [11]. For example, the bacteria *Acidithiobacillus Ferrooxidans* has an optimal growth pH of 1.4, but its cytoplasmic pH is maintained around 6.4 through a pH homeostasis mechanism [2], [12]. Fortunately, the cytoplasm of a few acidophiles can be acidic and thus the proteins exist in their cytoplasm are presumably acidostable. Recently, the entire genome of such an acidophile (*Acetobacter aceti*) was sequenced [13], making it possible to perform a systematic study to uncover the factors governing acidostability.

In this study, we attempt to develop a scoring function for discriminating proteins from bacteria with acidic cytoplasm (AP) and those with neutral cytoplasm (NP) based on their sequences using an integrated statistical and machine learning method. The proteins from *Acetobacter aceti* are considered as APs because the bacteria not only can thrive in acidic environments but also has acidic cytoplasm. Proteins from five organisms that thrive in acidic environments but have near-neutral cytoplasmic pH are used as NP controls (Table 1). Such controls are chosen to reduce the possible influence of pH homeostasis. To the best of our knowledge, this work represents the first systematic comparative study of the AP/NP orthologs.

**Table 1:** The list of organisms whose proteins are used to generate the non-redundant AP-NP ortholog pairs.

Organism	pH_opt_	pH_in_	# of proteins
*Acidithiobacilus ferrooxidans* ATCC_23270	1.8	6.5	3147
*Acidithiobacilus ferrooxidans* ATCC_53993	1.8	6.5	2826
*Sulfolobus acidocaldarius* DSM 639	1.8	6.5	2224
*Sulfolobus solfataricus* P2	2.5	6.5	2978
*Thermoplasma acidophilum* DSM 1728	1.4	6.4	1484
*Acetobacter aceti* NBRC 14818	6.2–3.5	5.8–3.9	4033

The pH values are obtained from [2], [42].

pH_opt_: optimal growth pH value; pH_in_: cytoplasmic pH value.

In order to perform the comparative study, we derive a set of 889 features from protein sequences. It would be better to include both sequence and structure information for investigating the acidostability factors; the vast majority of proteins especially acidostable ones, however, have no solved structures. In addition, the basic dogma that protein amino acid primary sequence determines its native structure implies that the sequence information may be sufficient to discover the mechanisms of pH-dependent protein stabilization. In fact, sequence-only features have been successfully used in related areas such as protein thermostability [14], [15], [16], [17].

An amino acid residue substitution matrix may reveal different overall trends for different types of proteins. For example, several matrices have been developed for thermophilic and mesophilic proteins to uncover the factors governing thermostability [17], [18], [19], [20], [21], [22]. However, to the best of our knowledge, there is no such a matrix for AP and NP proteins. Thus, in this study we calculate each of the 380 types of amino acid residue substitutions based on the pairwise BLAST alignments of all AP-NP ortholog pairs and construct an acidostability substitution matrix.

## Methods

### Datasets

All protein sequences of six organisms (Table 1) were downloaded from the NCBI protein database (http://www.ncbi.nlm.nih.gov/). The names, optimal growth pH and cytoplasm pH of these six organisms are provided in Table 1. The proteins from *Acetobacter aceti* are considered as acidostable proteins (AP) and proteins from other organisms are used as non-acidostable proteins (NP). A list of non-redundant AP-NP ortholog pairs are obtained using an established procedure [17]. In brief, we firstly perform all-against-all BLAST searches [23] for all AP against NP sequences. The resulted homolog pairs between AP and NP sequences are filtered based on the following conservative criteria to identify putative orthologs:

1. Reciprocal best BLAST hits with *e*-value in BLAST searches less than 10^−10^;
2. The difference of two sequences is less than 5% of the shorter sequence;
3. Higher than 30% sequence similarity.

**Figure 1:**
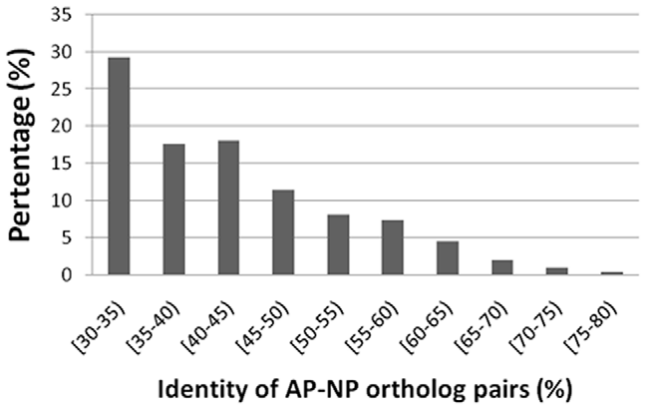
Protein sequence identity distribution between the AP-NP ortholog pairs. The x-axis is the protein identity of AP-NP ortholog pairs. For example, [30–35) means 30≤ identity<35. The y-axis is the corresponding percentage of proteins in the particular identity region.

Secondly, we remove all transmembrane proteins, predicted by TMHMM 2.0 (http://www.cbs.dtu.dk/services/TMHMM/) because transmembrane and global proteins may use different mechanisms to survive in acidic environments. In addition, to avoid the statistical bias caused by AP-NP pairs with similar sequences, the blastclust program [23] is used to remove sequence redundancy. The minimum length coverage of blastclust clustering is set to 0.5 and the sequence similarity threshold was 0.25. As the result of these selection steps, the two amino acid sequences of an AP-NP pair is very similar (i.e. orthlogous) but they are distinct from sequences in other AP-NP pairs. Only protein sequences shorter than 600 and longer than 50 are included in the final non-redundant AP-NP ortholog dataset. The final dataset includes 393 AP-NP ortholog pairs. The protein sequence identity distribution between the AP-NP ortholog pairs is shown in Figure 1. The sequences and accession numbers of these proteins are available in the supplementary materials File S1.fa and File S2.fa.

### Amino Acid Substitution Matrix

We calculate each of the 380 types of amino acid residue substitution based on the BLAST alignment results of all AP-NP protein ortholog pairs. The NP to AP amino acid residue substitution is considered as forward direction and AP to NP is considered as reverse direction. The statistical significance of forward and reverse substitutions are estimated using the two-sided Fisher’s exact test [17].

### Features

A set of 889 features derived from protein sequences, calculated with various software programs or in-house scripts, are used to encode each protein sequence (Table 2). These features include the absolute counts of amino acids and other properties (denoted as *c_k_*) and those normalized by chain length (labeled as *x_k_*). A number of programs, including ProtParam [24], NetSurfP [25] and disEMBL [26], are used to predict the proteins’ structural properties such as solvent accessible surface area(ASA) [27], [28], exposed/buried residues [29] and secondary structure [30], [31]. Although the cellular localization of proteins may play a role in protein acidostability and algorithms for predicting localization are available [32], [33], we do not use the information because the two orthologous proteins in each pair likely share the same localization.

**Table 2:** The list of the 889 sequence based features.

Protein feature	# of Features	Source
Sequence length (L)	1	In house script
Number and composition of amino acids	40	
Number and composition of dipeptides	800	
Number and percentage of positive, negative and all charged residues, as well as the net charges	8	
Number and percentage of small (T and D), tiny (G, A, S and P), aromatic (F, H, Y, W), aliphatic, hydrophobicand polar residues	12	
Number and percentage of residues which can form hydrogen bond in side chain	2	
Number of sulfide atoms	1	
The average of the maximum solvent accessible surface area (ASA) of each amino acid	1	Eisenhaber [28]
Predicted isoelectric point (pI) of the protein and the average pI on all residues (pIa)	2	ProtParam [24]
Instability index and instability class	2	
Aliphatic index	1	
Gravy hydropathy index	1	
Number and Composition of the predicted secondary structure residues	6	NetSurfP [25]
Number and Predicted percentages of buried/exposed residues	4	
The overall length and percentage of all coils, rem465, and hot loop	6	disEMBL [26]
Mean Relative Surface Accessibility – RSA	1	
Mean Z-fit score for RSA prediction	1	

### Scoring Function

For each AP-NP protein pair, the relative feature difference Δ*x_i_* is calculated using the following formula:
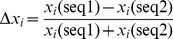
(3)Where *x*_i_(seq1) and *x*_i_(seq2) are the values of the *i* th features from the first sequence and the second sequence, respectively. We construct a scoring function by a linear combination of the ten most important features. The scoring function can be written as
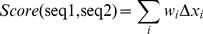
(4)where 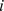 runs over all of the 10 most important features and 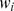
is the weight of each feature. A hill-climbing algorithm is used to fit the weights of all these features [17], [34]. All weights are optimized with the absolute values limited between 0 and 1. The initial value of each weight is assigned randomly. The weights are randomly updated and the number of correct predictions is recorded. The new weights are kept if the number of the correctly predicted ortholog pairs increases, or else the weights are rolled back to the previous values. The optimization is repeated 108 times and the weight which maximized the number of positive score values are recorded.

### Random Forest

Random Forest (RF) algorithm [35] is an ensemble classification or regression method using the consensus of many decision trees. Each of the member trees is built on a bootstrap sample from the training data and utilizes a random subset of available variables. It is robust and particularly suitable for classifying high-dimensional and noisy data. Besides conventional classification or regression, an important application of RF is that it can assess the importance of various features based on their contributions to the performance of the predictive models. Gini importance is frequently used as a metric for measuring the relative importance of attributes; it is calculated as the summation of the Gini impurity decreases in node splits made on the feature over all trees. The Gini impurity is defined as:
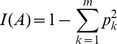
(1)where *k* = 1,2,…,*m* are possible classes and *p_k_* is the relative frequency of class *k* in a node A. *I(*A*)* is equal to zero when all cases in the node belong to a single class and reaches its maximum when cases are equally distributed to all classes.

In this study, an R implementation of the RF algorithm (http://cran.r-project.org/web/packages/randomForest/index.html) is used to construct the predictive model. Each model built in the study consists of 5,000 decision trees.

### Performance Assessment

Several metrics are used to estimate the performance of the models developed in the study. Since the discrimination of AP and NP proteins is treated as a binary classification problem, we plot the receiver operation characteristic (ROC) curve based on the prediction results of AP and NP sequences. ROC is a graphic plot of the true-positive rate (sensitivity) against the false-positive rate (1-specificity). The area under an ROC curve (AUC) represents the trade-off between sensitivity and specificity. AUC is in the range of 0 to 1 and the bigger the AUC, the better the performance. An AUC of 0.5 indicates random classification. The accuracy of a classification is calculated by.
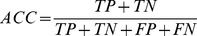
(2)where TP, TN, FP and FN are the numbers of true positives, true negatives, false positives and false negatives, respectively. A true case stands for the AP protein is correctly identified.

## Results and Discussion

In this study, we first construct a non-redundant dataset which contains 393 AP-NP ortholog protein pairs. The amino acid residue compositions of both groups of proteins are analyzed and an amino acid residue substitution matrix is generated to assess the substitution preference in the AP-NP pairs. We then generate a set of 889 features to decode AP-NP proteins and use the Random Forest algorithm to rank the relative importance of these features for discriminating APs and NPs. A scoring function is developed using the 10 most important features. Finally, RF models are developed using all or selected features.

### Amino Acid Composition

We calculate the amino acid compositions of all AP-NP ortholog protein pairs and evaluate the statistical difference using the paired and unpaired *t*-test (Table 3). It can be seen in the Table 3 that the most significantly increased residues in APs are Ala (A), Pro (P) and Thr (T) while residues reduced in number include Ile (I), Asn (N), Gln (Q) and Tyr (Y). The results are largely consistent with current understanding of protein acidostabilization. For example, Pro has a rigid five-member ring which can often increase protein stability. The side chain of both Asn and Gln contain an amide which is labile to hydrolysis in acidic conditions [36]. Thus the reduction of Asn and Gln can improve the acidostability of a protein. Interestingly, the percentage of His in NP is higher than in AP (paired *t*-test *p*-value = 2.48×10^−3^), suggesting metal ions are unlikely a significant factor, at least for *Acetobacter aceti*, in protein acidostabilization.

**Table 3:** Comparison of the amino acid composition of AP-NP ortholog pairs.

Amino Acid	Composition in AP	Composition in NP	*P*-value (*t*-test)	*P*-value (paired*t*-test)
**S**	**0.053±0.015**	**0.051±0.016**	**0.02**	**5.29×10** ^−**03**^
*Q*	*0.030±0.012*	*0.036±0.016*	*3.15×10* ^−*8*^	*1.32×10* ^−*10*^
*N*	*0.025±0.011*	*0.029±0.015*	*1.93×10* ^−*4*^	*4.10×10* ^−*6*^
**T**	**0.056±0.015**	**0.046±0.014**	**2.70×10** ^−**21**^	**8.01×10** ^−**26**^
C	0.010±0.009	0.010±0.010	0.20	0.02
G	0.086±0.021	0.083±0.022	0.16	0.02
**A**	**0.114±0.030**	**0.106±0.037**	**8.40×10** ^−**4**^	**3.75×10** ^−**6**^
*H*	*0.022±0.011*	*0.024±0.012*	*0.02*	*2.48×10* ^−*3*^
M	0.026±0.011	0.025±0.011	0.45	0.31
*Y*	*0.019±0.011*	*0.027±0.014*	*1.37×10* ^−*17*^	*3.38×10* ^−*29*^
F	0.032±0.012	0.032±0.014	0.61	0.44
V	0.077±0.019	0.076±0.020	0.25	0.11
L	0.096±0.023	0.098±0.027	0.13	0.02
**P**	**0.051±0.016**	**0.048±0.016**	**3.63×10** ^−**3**^	**1.82×10** ^−**5**^
*I*	*0.052±0.016*	*0.059±0.023*	*7.49×10* ^−*6*^	*1.14×10* ^−*7*^
W	0.010±0.008	0.011±0.010	0.11	0.01
**D**	**0.059±0.015**	**0.056±0.015**	**0.02**	**1.75×10** ^−**3**^
E	0.065±0.019	0.066±0.020	0.46	0.26
K	0.040±0.022	0.043±0.029	0.14	0.04
R	0.074±0.024	0.073±0.024	0.94	0.89

The *p*-values based on *t*-test and paired *t*-test. Amino acids significantly increased or reduced in AP than NP are in bold or italics, respectively.

Although we calculate the *p*-values of both paired and unpaired *t*-tests, the paired one should be considered as a better choice for estimating the significance of the composition difference. The two methods differ at the level of information granules. The former is at the overall amino acid composition differences. We focused on the differences while the latter instead is on AP and NP ortholog pairs. Thus, the paired approach may reduce or eliminate the effects of confounding factors such as protein families because it is well established that the amino acid composition may vary in different protein classes [37]. In addition, a protein level study may be more relevant to designing acidostable proteins because orthologs are essentially mutants with multiple mutations.

### Amino Acid Residue Substitution Matrix

The pairwise amino acid residue substitutions are calculated from the pairwise sequence alignment of AP-NP pairs and displayed in Table 4. We calculate the absolute counts and the ratio of each substitution to the opposite replacement. Significantly biased substitutions (*p*-value <10^−10^, two-sided Fisher’s exact test) are shown in bold and color. Red cells represent substitutions favored in the forward direction (NP to AP), while blues are favored in the reverse direction (AP to NP). Overall, there are 81 significantly biased substitutions which include 43 favored in the forward direction and 38 favored in the opposite direction. Specifically, Gln (Q) is preferred to be substituted by Glu (E), Arg (R) and Ala (A) in the forward direction so the acid-labile amide is reduced. For the same reason, Asn (N) is preferred to be replaced by Asp (D), Gly (G) and Ala (A). Other notable preferred substitutions in the forward direction include Tyr and Ile by Leu, Lys by Arg, Ser by Ala, etc. In the reverse direction (AP to NP) substitution, Cys and Thr are preferred to be substituted by Ala and Val, Met by Leu, and Arg by Lys, etc. Most of the substitution preferences are asymmetrical. The ratios in this matrix may reflect the acidostable adaption induced substitution biases and should be useful in designing acidostable proteins.

**Table 4:** Amino acid substitution matrix between AP and NP proteins.

NP/AP	Uncharged polar				Nonpolar												Charged			
	S	Q	N	T	C	G	A	H	M	Y	F	V	L	P	I	W	D	E	K	R
S	1761	164	199	448	*45*	288	*681*	102	45	42	39	129	126	183	80	18	280	283	177	**251**
	–	1.50	1.21	0.82	*0.70*	0.92	*0.90*	1.42	1.15	1.91	1.15	0.97	1.18	0.97	1.45	1.64	1.28	1.14	1.42	**1.34**
Q	109	945	82	100	6	101	*211*	117	47	42	32	88	*146*	51	54	19	148	*360*	167	305
	0.66	–	0.85	0.66	0.38	0.71	*0.61*	1.00	0.73	1.91	0.94	0.86	*0.89*	0.66	1.20	1.06	0.65	*0.91*	0.82	0.81
N	165	97	1144	89	10	*151*	*130*	93	14	35	18	41	63	48	30	10	212	108	73	*128*
	0.83	1.18	–	0.55	0.59	*0.74*	*0.65*	1.39	0.58	1.67	0.62	0.98	0.98	0.83	0.88	1.43	0.71	0.81	0.72	*0.94*
T	549	151	161	1908	49	**175**	**488**	95	71	68	67	**368**	**258**	139	198	28	211	**264**	160	**286**
	1.23	1.51	1.81	–	1.00	**1.17**	**1.16**	1.70	0.95	2.34	1.14	**1.26**	**1.24**	1.14	1.78	2.80	1.45	**1.52**	1.31	**1.69**
C	**64**	16	17	49	411	27	**160**	12	23	11	20	**130**	63	12	**54**	7	7	6	6	**27**
	**1.42**	2.67	1.70	1.00	–	1.04	**1.32**	1.71	1.00	1.10	1.82	**1.55**	0.98	0.80	**1.69**	0.70	0.37	0.60	0.67	**1.13**
G	313	142	**203**	150	26	6036	**747**	**87**	44	44	43	83	107	153	50	35	254	194	130	219
	1.09	1.41	**1.34**	0.86	0.96	–	**1.05**	**1.14**	1.22	3.67	0.93	1.05	0.96	1.02	1.56	1.94	0.95	0.97	0.99	1.18
A	**753**	**344**	**199**	*420*	*121*	710	4986	**169**	**165**	161	**127**	733	552	*364*	299	**64**	335	561	**293**	528
	**1.11**	**1.63**	**1.53**	*0.86*	*0.76*	0.95	–	**1.41**	**1.26**	2.40	**1.13**	1.19	1.22	*0.89*	1.29	**1.60**	0.96	1.01	**1.20**	1.13
H	72	117	67	56	7	*76*	*120*	951	19	135	59	48	**90**	53	26	19	86	95	83	*157*
	0.71	1.00	0.72	0.59	0.58	*0.87*	*0.71*	–	0.83	2.14	1.09	0.79	**1.22**	1.02	0.84	1.19	0.71	0.81	1.36	*0.78*
M	39	64	24	75	23	36	*131*	23	775	49	93	**192**	**499**	17	**220**	31	24	53	37	**77**
	0.87	1.36	1.71	1.06	1.00	0.82	*0.79*	1.21	–	1.96	1.15	**1.05**	**1.11**	0.57	**1.14**	1.82	1.26	1.13	1.12	**1.20**
Y	22	22	21	29	10	12	67	*63*	25	1134	263	61	111	16	55	*42*	18	29	14	54
	0.52	0.52	0.60	0.43	0.91	0.27	0.42	*0.47*	0.51	–	0.77	0.46	0.53	0.46	0.76	*0.39*	0.47	0.43	0.40	0.53
F	34	34	29	59	11	46	*112*	54	81	343	1541	*185*	430	22	**178**	85	21	30	21	46
	0.87	1.06	1.61	0.88	0.55	1.07	*0.88*	0.92	0.87	1.30	–	*0.94*	0.99	0.81	**1.24**	0.87	0.72	0.88	0.88	0.64
V	133	102	42	*293*	*84*	79	616	61	*182*	133	**197**	3271	**1062**	119	1264	48	66	147	103	173
	1.03	1.16	1.02	*0.80*	*0.65*	0.95	0.84	1.27	*0.95*	2.18	**1.06**	–	**1.22**	0.93	1.19	1.66	0.78	1.04	1.17	0.97
L	107	**164**	64	*208*	64	112	454	*74*	*448*	**209**	433	*872*	5028	108	**1100**	**110**	75	131	101	246
	0.85	**1.12**	1.02	*0.81*	1.02	1.05	0.82	*0.82*	*0.90*	**1.88**	1.01	*0.82*	–	0.93	**1.08**	**1.28**	0.85	0.81	1.15	0.84
P	189	77	58	122	15	150	**407**	52	30	35	27	128	116	2930	67	14	176	212	120	153
	1.03	1.51	1.21	0.88	1.25	0.98	**1.12**	0.98	1.76	2.19	1.23	1.08	1.07	–	2.03	1.40	1.20	1.20	1.11	1.20
I	55	45	34	111	*32*	32	231	31	*193*	72	*144*	1065	*1022*	33	2214	28	26	57	39	97
	0.69	0.83	1.13	0.56	*0.59*	0.64	0.77	1.19	*0.88*	1.31	*0.81*	0.84	*0.93*	0.49	–	1.12	0.65	0.77	0.81	0.78
W	11	18	7	10	10	18	*40*	16	17	**109**	**98**	29	*86*	10	25	384	8	17	19	30
	0.61	0.95	0.70	0.36	1.43	0.51	*0.63*	0.84	0.55	**2.60**	**1.15**	0.60	*0.78*	0.71	0.89	–	0.36	0.65	0.90	0.63
D	219	226	299	146	19	266	350	121	19	38	29	85	88	147	40	22	2923	754	154	217
	0.78	1.53	1.41	0.69	2.71	1.05	1.04	1.41	0.79	2.11	1.38	1.29	1.17	0.84	1.54	2.75	–	1.11	0.90	1.09
E	249	**396**	134	174	10	201	554	118	47	67	34	141	161	176	74	26	682	2830	263	431
	0.88	**1.10**	1.24	0.66	1.67	1.04	0.99	1.24	0.89	2.31	1.13	0.96	1.23	0.83	1.30	1.53	0.90	–	1.12	1.09
K	125	203	102	122	9	131	*245*	61	33	35	24	88	88	108	48	21	171	235	1453	*576*
	0.71	1.22	1.40	0.76	1.50	1.01	*0.84*	0.73	0.89	2.50	1.14	0.85	0.87	0.90	1.23	1.11	1.11	0.89	–	*0.87*
R	*187*	**378**	**136**	*169*	*24*	185	466	**201**	*64*	102	72	178	293	127	124	48	200	396	**662**	3400
	*0.75*	**1.24**	**1.06**	*0.59*	*0.89*	0.84	0.88	**1.28**	*0.83*	1.89	1.57	1.03	1.19	0.83	1.28	1.60	0.92	0.92	**1.15**	–

The top number in each cell (begin with amino acid residue) is the observed substitution instances and bottom one is the ratio of the number of the substitution cases to the opposite substitution. Significant biased substitutions (*p*-value <10^−10^, two-sided Fisher’s exact test) are highlighted in bold and italics. Bold cells are significant AP favored substitutions while italics cells are NP favored.

**Figure 2:**
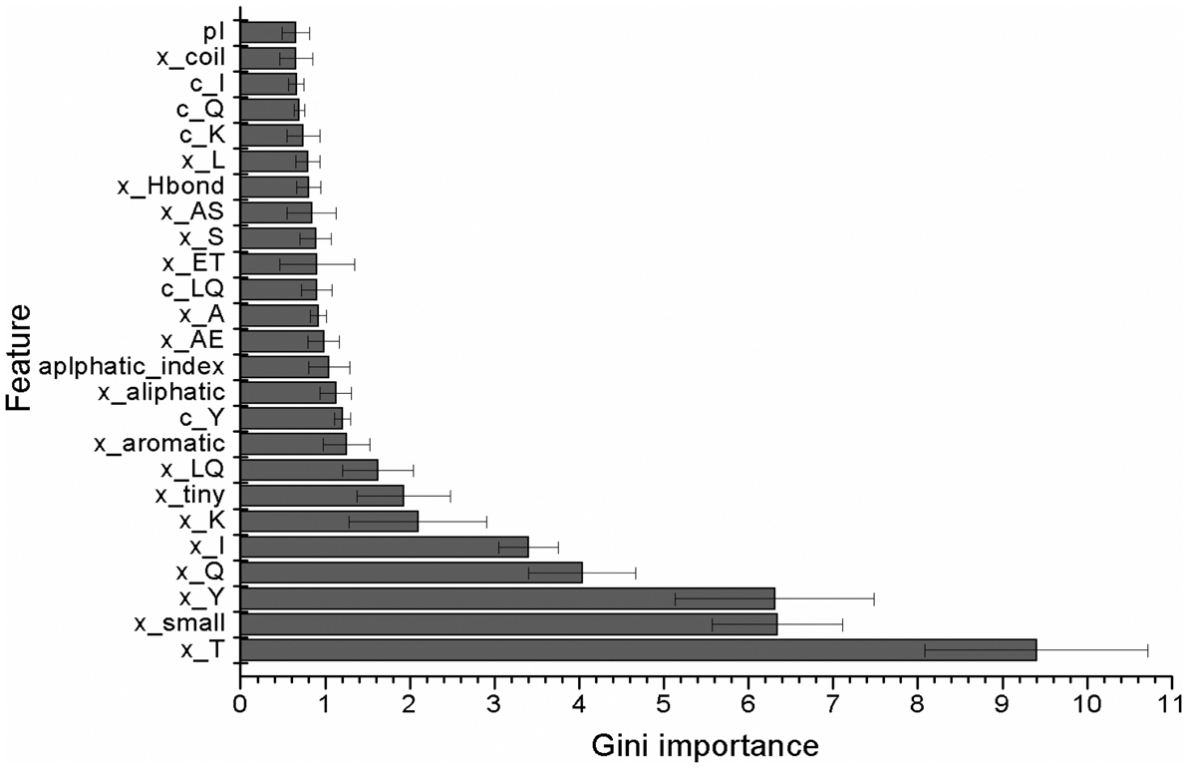
The 25 most important features ranked by the Gini importance. The prefix x_ and c_ indicate the normalized and absolute features. Single letter amino acid code is used. For example, x_A is the normalized ratio of Alanine residue and x_AS is the normalized ratio of dipeptide AS (Alanine-Serine). Coil: residues in coils: Hbond: residues which can form hydrogen bond in side chain; aliphatic, aromatic, tiny and small are aliphatic, aromatic, tiny and small residues respectively.

**Figure 3:**
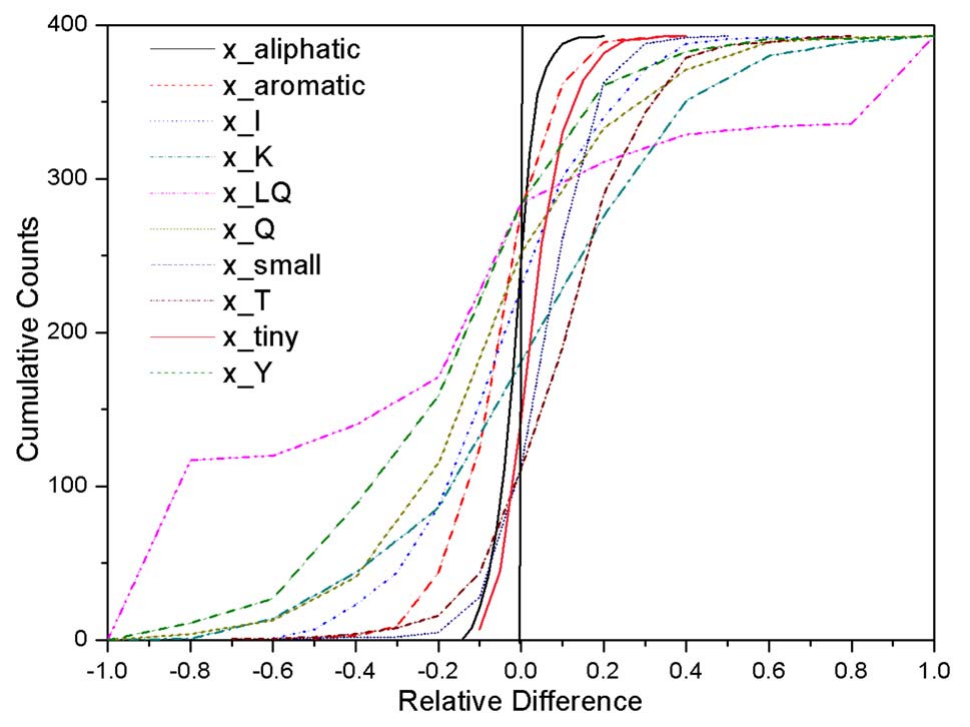
The cumulative curves of 10 most important normalized features against the relative difference between the AP and NP sequence. The prefix x indicates normalized features. Single letter amino acid code is used. For example, x_Y is the ratio of Tyrosine residue and x_LQ is the normalized ratio of dipeptide LQ (Leucine-Glutamine). Aliphatic, aromatic, small and tiny are aliphatic, aromatic, small and tiny residues, respectively.

**Table 5:** The weights of 10 features used in the scoring function.

Feature	x_K	x_small	x_T	x_tiny	x_aliphatic	x_aromatic	x_I	x_LQ	x_Q	x_Y
Weigth	0.68	0.75	0.90	0.58	−0.97	−0.74	−0.96	−0.04	−0.01	−0.53

The prefix x indicates the normalized features.

### Relative Importance of Features

We rank all the 889 features derived from protein sequence using the RF algorithm. Five-fold cross validation is used to evaluate the relative importance of features. The set of 393 AP-NP protein pairs are randomly split into 5 groups with approximately equal sample size. Four groups are used as training set and the remaining group is reserved for testing. We build a RF model with 5,000 trees for the first training set. The procedures are then repeated four more times until each group has been used as a testing set, once. The average and standard deviation of the Gini importance of top 25 features (top25) are displayed in Figure 2. We find that the normalized features are generally more important than the absolute counts of the corresponding features and the normalized amino acid residues composition of Thr, Tyr, Gln, Ile and Lys are among the most important. Both small and tiny categories are among the top 25 features (Figure 2), suggesting that the volume of amino acids may play a role in protein acidostability. In addition, Figure 2 indicates that both aromatic and aliphatic residues are important in discriminating AP and NP proteins.

**Figure 4:**
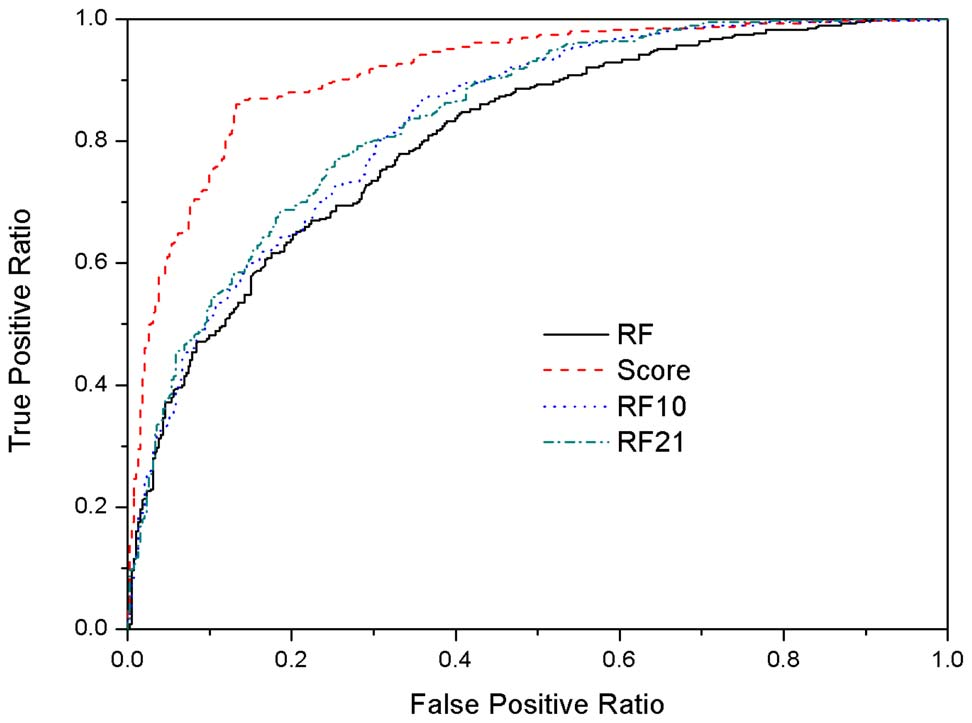
The ROC curves. RF: a RF model based on all features; RF10: a RF model using 10 most important features; RF21 using 21 features selected by varSelRF package [41]; Score: based on the score function.

### Scoring Function

The cumulative curves of the relative feature difference of the 10 most important normalized features in the training dataset are shown in Figure 3. The cumulative curves show typical sigmoid shapes, the inflexion points of which are located at the half height. The sign of the weight of each feature is determined by the location of its inflexion point of the cumulative curve: positive for features located to the left and negative for those to the right of the zero difference line. Thus the signs of x_T, x_small, x_K and x_tiny are positive and the ones for x_Y, x_Q, x_I, x_LQ, x_aromatic and x_aliphatic are negative. The features of x_aromatic and amino acid Tyr (Y) indicate aromatic amino acid is important to distinguish the AP and NP proteins. It is because the aromatic ring in such amino acid may form pi stacking and cation-pi bond which can improve the stability of proteins in acid environment [38]. The aliphatic amino acids are hydrophobic and can have hydrophobic interactions which can increase the stabilization of proteins [39]. The features of x_small and x_tiny indicate the amino acid volume has contribution in distinguishing the AP and NP proteins. It may be the reason that amino acid with small volumes can contribute the dense packing interaction of proteins [39], [40] which can enhance the stabilization of proteins.

To determine whether the optimization is trapped in a local maximum, the optimization is repeated four more times using different random initial values. The results are very close to each other. The average value of each weight is used as the final weights (Table 5). It is noteworthy that the absolute values of weights do not reflect the relative importance in the ability of discrimination, because the features are not normalized. The signs of the weight indicate these features are favorable to AP (+) or not (−).

We use the scoring function to discriminate the AP and NP sequences. It can correctly discriminate 338±1.2 out of 393 ortholog pairs (86.0% ACC). To further evaluate the ability of the scoring function in discriminating AP-NP pairs, we challenge the scoring function in discriminating non-homologous AP-NP pairs. In this test, we compare each AP protein in AP to all NP proteins. The overall accuracy of these 393×393 pairwise comparisons is 76.65%, indicating that the scoring function can be able to discriminate non homologous AP-NP pairs as well. The result suggests that the AP sequences share common acidostabilization mechanisms, resulting in sufficient acidostability for acidic conditions.

### Random Forest Classification Models

We first use a standard five-fold cross validation procedure to estimate the performance of Random Forest classification models using all 889 features. The AUC and ACC are 0.805 and 72.3% (Figure 4), respectively. The AUC and ACC are improved to 0.830 and 73.6%, respectively, for models using only top 10 features, ranked using Gini importance. In addition, we use varSelRF package [41] to identify the best combination feature set of 21 features. The AUC and ACC of RF models based on 21 features are 0.837 and 75.3%, respectively. The score function achieves an AUC of 0.913, indicating that the score function achieves the best performance. The improved performance using selected features suggests that the top features are important to protein acidostability, which can be used as a general guide for designing acidostable proteins. For example, the number of lysine, tyrosine, and small or tiny residues should be increased at the cost of aliphatic and aromatic residues and glutamine, in particular.

### Conclusion

In this work, we develop a scoring function and Random Forest predictive models for discriminating acidostable and non-acidostable proteins. The scoring function and models are capable of discriminating both AP-NP ortholog proteins and non-ortholog proteins. The analysis of amino acid composition and residue substitution preference between AP and NP clearly indicates that different amino acid residues may contribute differently to the protein acidostability. The overall trends of acidostabilization uncovered in the study should be useful for designing acidostable proteins.

## Supporting Information

File S1 **AP sequences in FASTA format.**
(FA) Click here for additional data file.

File S2 **NP sequences in FASTA format.**
(FA) Click here for additional data file.
